# Construction of 1D SnO_2_-coated ZnO nanowire heterojunction for their improved n-butylamine sensing performances

**DOI:** 10.1038/srep35079

**Published:** 2016-10-13

**Authors:** Liwei Wang, Jintao Li, Yinghui Wang, Kefu Yu, Xingying Tang, Yuanyuan Zhang, Shaopeng Wang, Chaoshuai Wei

**Affiliations:** 1 School of Marine Sciences, Guangxi University, Nanning, 530004, China; 2 Coral Reef Research Center of China, Guangxi University, Nanning, 530004, China; 3 Experimental Practising & Teaching center, Hebei GEO University, Shijiazhuang, 050031, China

## Abstract

One-dimensional (1D) SnO_2_-coated ZnO nanowire (SnO_2_/ZnO NW) N-N heterojunctions were successfully constructed by an effective solvothermal treatment followed with calcination at 400 °C. The obtained samples were characterized by means of XRD, SEM, TEM, Scanning TEM coupled with EDS and XPS analysis, which confirmed that the outer layers of N-type SnO_2_ nanoparticles (*avg.* 4 nm) were uniformly distributed onto our pre-synthesized n-type ZnO nanowire supports (diameter 80~100 nm, length 12~16 μm). Comparisons of the gas sensing performances among pure SnO_2_, pure ZnO NW and the as-fabricated SnO_2_/ZnO NW heterojunctions revealed that after modification, SnO_2_/ZnO NW based sensor exhibited remarkably improved response, fast response and recovery speeds, good selectivity and excellent reproducibility to n-butylamine gas, indicating it can be used as promising candidates for high-performance organic amine sensors. The enhanced gas-sensing behavior should be attributed to the unique 1D wire-like morphology of ZnO support, the small size effect of SnO_2_ nanoparticles, and the semiconductor depletion layer model induced by the strong interfacial interaction between SnO_2_ and ZnO of the heterojunctions. The as-prepared SnO_2_/ZnO NW heterojunctions may also supply other novel applications in the fields like photocatalysis, lithium-ion batteries, waste water purification, and so on.

As one kind of the important organic amines, n-butylamine is widely used as marker compounds in medical diagnosis and food industries to control the qualities, or a chemical intermediate for producing emulsifying agents, tanning agents, special soaps and rubber chemicals. Furthermore, n-butylamine is also utilized in the production of polymer industries, dyes, textiles, insecticides and pharmaceuticals^1^,^2^. However, it’s toxicity, volatility and easy absorption through skins can lead to skin, upper respiratory tract and eye irritation if people are directly exposed to n-butylamine vapor^3^,^4^. Therefore, the effective and rapid detection of n-butylamine in surrounding environment is quite necessary and of great realistic significance to serve our daily lives.

Although several methods such as GC-MS spectrometry and colorimetric methods have been applied to detect n-butylamine^5^, there are still some shortcomings such as the expensive and complicated equipment, the complex pretreatment, the time-consuming subsequent analytical procedures and so on, all of which hinder their convenient and real-time applications. Thus, it’s an urgent need to develop fast, online and selective n-butylamine sensors to benefit human beings^6^.

Currently, chemiresistive gas sensors have gotten sufficient development due to their easy handling, low cost and real-time detection advantages^7^,^8^. Among the vast majority of sensing materials, nanostructured semiconducting metal oxides (SMOs), *e.g.* Nb_2_O_5_^9^, SnO_2_^10^, α-Fe_2_O_3_^11^, ZnO^12^, In_2_O_3_^13^, WO_3_^14^ and CuO^15^ etc., have exhibited potential superiorities in terms of simple preparation, good compatibility, fast response and recovery, and fine stability. But to date, there were still few reports on SMO-based sensors for the detection of n-butylamine, and the responses of the reported V_2_O_5_, AgV_x_O_y_ and WO_3_ nanostructured sensors were too low to meet the practical requirements^16^,^17^,^18^. So there still needs further improvements for the SMO-based sensors.

In order to improve their performances, SMO hetero-nanostructures consisting of two or more composites with multiple functions have gained more attention, such as CuO/SnO_2_ nanorods^19^, ZnO/SnO_2_ hollow spheres^20^, SnO_2_/ZnO nanowire^21^, SnO_2_/α-Fe_2_O_3_ nanotube^22^, ZnO/Co3O4 composite^23^, ZnO/CuO heterojunction^24^, WO_3_/TiO_2_ hybrid^25^ and so on. The sensing mechanism can be explained by the change of the interfacial resistance due to the formation of a heterojunction^26^. Among various SMOs, SnO_2_ and ZnO (*E*g = 3.6 and 3.37 eV at 300 K, respectively) are the most promising sensor candidates for high-performance VOCs sensors. After combining SnO_2_ with ZnO, the heterojunction nanostructures can greatly improve their gas-sensing properties^27^,^28^,^29^. Besides, due to the high surface-to-volume ratio and fast electron transportation properties, one dimensional (1D) ZnO nanowires (NWs) can effectively promote the diffusion of gases through the devices, so the responses and reaction speeds can be greatly enhanced^30^,^31^. However, as far as we know, reports on employing 1D SnO_2_/ZnO nanowire heterojunctions for n-butylamine sensor application were still fewer^32^,^33^. It should be expected that such sensor will have an excellent prospect in detecting n-butylamine.

In this work, by combining the ideas on surface modification and junction formation^34^,^35^,^36^,^37^, 1D SnO_2_ coated ZnO NW N-N junctions have been formed via a two-step solution reaction. The ZnO NWs, which were pre-synthesized by a hydrothermal method with diameters of 80~100 nm and lengths of 12~16 μm^31^, were further used as supports to load SnO_2_ nanoparticles with average size of 4 nm and construct SnO_2_/ZnO heterojunctions, whose growth process was illustrated. The gas sensor based on such heterojunction was applied to detect several VOCs, and exhibited higher response and faster response and recovery speeds compared with the pristine ZnO NW and SnO_2_ based sensors, especially good selectivity and reproducibility to n-butylamine. Our results imply that 1D SnO_2_/ZnO NW heterojunctions may also provide other potential applications in the future. The sensing mechanisms were discussed to help explain such improved sensing properties.

## Results

### Characterization

**Figure 1:**
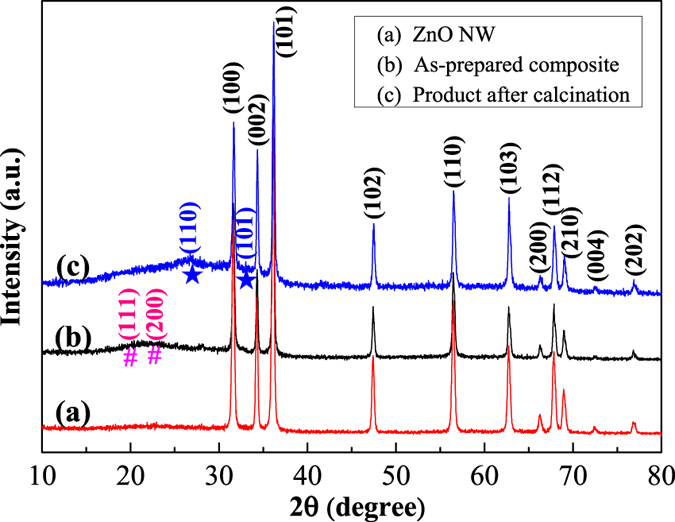
XRD pattern of (**a**) the pristine ZnO support, (**b**) the as-prepared ZnSn(OH)_6_/ZnO composite, and (**c**) the obtained SnO_2_/ZnO heterojunction.

In this work, the as-prepared SnO_2_/ZnO heterojunction materials were firstly characterized to confirm the structure and composition, and then assembled to be gas sensors to check their sensing performances. The crystalline phases of the samples were identified by powder X-ray diffraction. Figure 1a–c shows the XRD patterns of the products prepared via hydrothermal/solvothermal methods and calcination treatment. All the dominant diffraction peaks in Fig. 1a–c can be ascribed to typical hexagonal wurtzite ZnO (JCPDS No. 36-1451) phase^26^,^31^. It means that the pristine ZnO support was successfully synthesized through one-step hydrothermal process but without annealing, as can be seen in Fig. 1a. However, in Fig. 1b, faint diffraction peaks from the hexagonal ZnO spectrum can be observed at 2θ of around 19.62° and 22.72°, which are accordant with the (111) and (200) planes of standard ZnSn(OH)_6_ phase (JCPDS No. 20-1455)^20^. This indicates that at the solvothermal stage, ZnSn(OH)_6_ has been formed peripherally at the expense of ZnO supports at 120 °C. Besides, two weak diffraction peaks centered at about 2θ = 26.61 and 2θ = 33.89, respectively, were also detected in the sample after calcination in Fig. 1c, which correspond to the (110) and (101) planes of tetragonal SnO_2_ (JCPDS, 41-1445)^20^, revealing the simultaneous decomposition of ZnSn(OH)_6_ and presence of SnO_2_ phase in the as-obtained SnO_2_/ZnO products, whose chemical compositions were further characterized by the following EDS.

**Figure 2:**
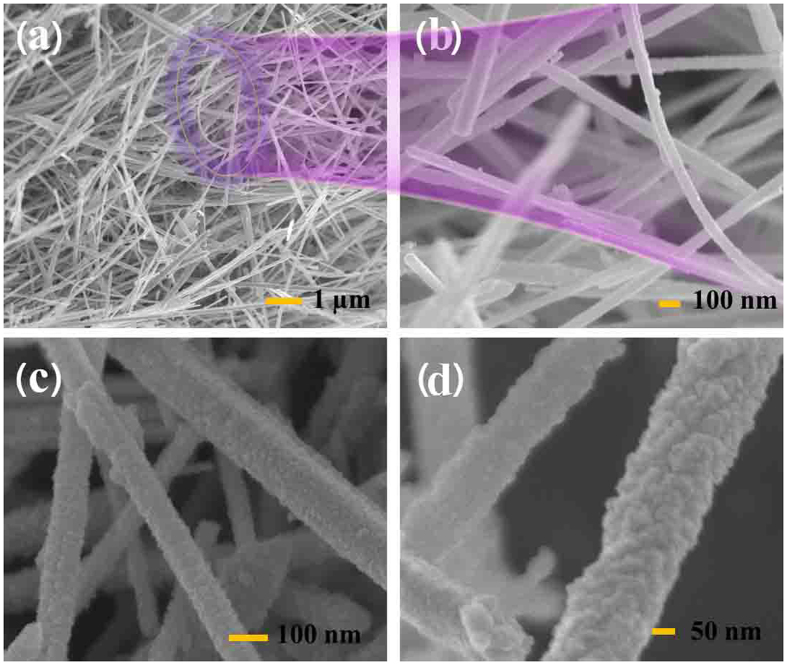
(**a,b**) The low and high magnified SEM images of the pristine ZnO NWs, SEM images of (**c**) ZnSn(OH)_6_/ZnO composite and (**d**) SnO_2_/ZnO heterojunction.

The size and morphology of the samples were characterized by field emission SEM in Fig. 2. Figure 2a,b show the low and high magnified FESEM images of the as-synthesized pristine ZnO support, which indicate that the samples were composed of large numbers of nanowires (diameter 80~100 nm, length 12~16 μm), and the smooth surfaces can be observed from the magnified image in Fig. 2b. The SEM image of ZnSn(OH)_6_/ZnO composite can be seen in Fig. 2c, where the surface morphology of the composite appears to be rough due to the solvothermal formation of ZnSn(OH)_6_ on the outer layers at the expense of ZnO supports. Figure 2d shows the SEM image of the SnO_2_/ZnO heterojunction. Apparently, the ZnO NWs were uniformly coated with layers of SnO_2_ nanoparticles (*avg.* 4 nm) with thickness of 10–20 nm. Although calcination at 400 °C for 2 h was applied to the samples, the wire-like morphology was still retained.

**Figure 3:**
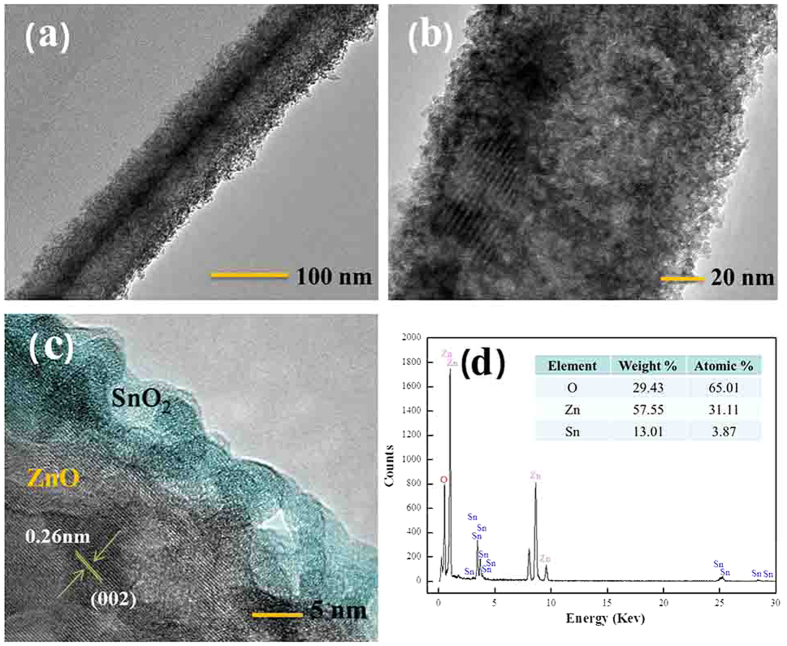
(**a**) TEM images of ZnSn(OH)_6_/ZnO composite, (**b,c**) TEM and HRTEM images of SnO_2_/ZnO heterojunction, (**d**) EDS spectrum of SnO_2_/ZnO heterojunction.

To further explore how the SnO_2_ nanocrystals coupled with the ZnO NWs, TEM and HRTEM analyses on the SnO_2_/ZnO composite were conducted. As presented in Fig. 3a, the TEM image clearly shows that the ZnSn(OH)_6_ precursor covers on the surfaces of ZnO product which maintains the general wire-like characteristics after solvothermal process. The TEM image in Fig. 3b displays the SnO_2_/ZnO composite after calcination, and the thickness of SnO_2_ layers is about 10–20 nm. The HRTEM image in Fig. 3c exhibits the interface region of a typical heterojunction, and the lattice fringes of the inner ZnO NW can be clearly observed, where the adjacent lattice plane of 0.26 nm is corresponding to the (002) plane lattice distance of hexagonal ZnO^31^. But the lattice fringes of the outer SnO_2_ nanocrystals with average size of 4 nm were dimmed, indicating the amorphous state which is coincident with the result in Fig. 1c. The above HRTEM observation clearly reveals that the SnO_2_ nanocrystals were successfully supported on the surface of the ZnO NWs, forming SnO_2_/ZnO interfaces. The good interfacial contact between SnO_2_ and hexagonal ZnO phases will result in an easier transfer of electrons from SnO_2_ to ZnO which is favorable for high gas-sensing performances. The EDS spectrum is applied in Fig. 3d to further identify the existence of O, Zn and Sn elements in the SnO_2_/ZnO composite.

**Figure 4:**
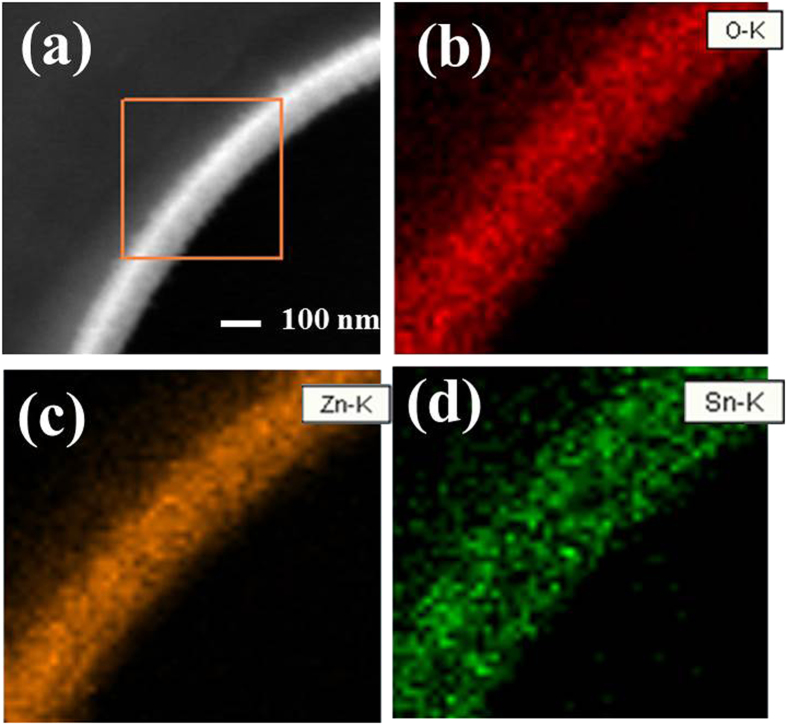
(**a**) A magnified TEM image of a typical SnO_2_/ZnO heterojunction, and the corresponding (**b**) O Kα1 map, (**c**) Zn Kα1 map and (**d**) Sn Kα1 map.

The SnO_2_-coated ZnO NW heterostructures were characterized by TEM phase mapping. Figure 4a exhibits a bright-field TEM image of a typical SnO_2_/ZnO nanowire heterojunction, where polycrystalline SnO_2_ and ZnO phases coexist. Figure 4b–d shows the elemental mapping images of O Kα1 (red), Zn Kα1 (brown) and Sn Kα1 (green), respectively, all of which take on the linear distribution thus proves that the SnO_2_ nanoparticles deposited uniformly along the ZnO NW support.

**Figure 5:**
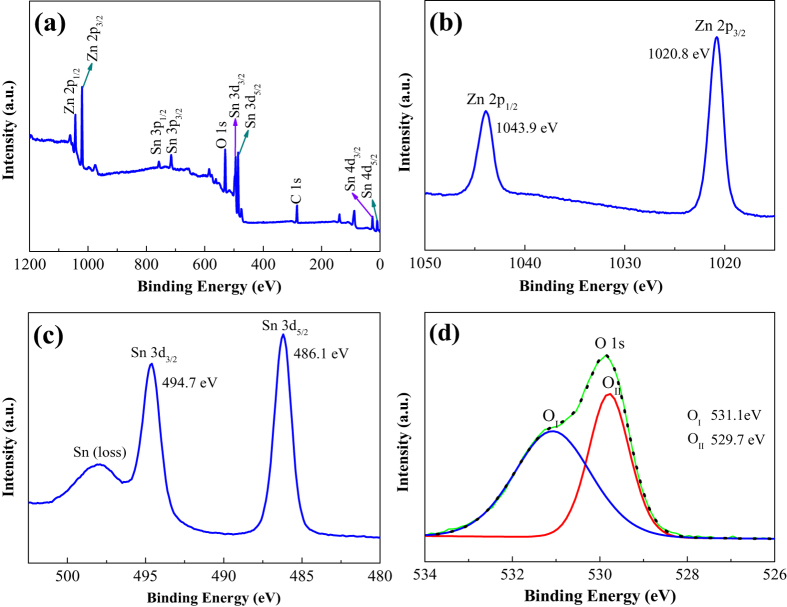
XPS spectra of the as-synthesized SnO_2_/ZnO heterojunction. (**a**) XPS full survey spectrum, (**b**) Zn 2p spectrum, (**c**) Sn 3d spectrum, (**d**) O 1s spectrum.

The surface compositions and their corresponding valence state of the as-synthesized SnO_2_/ZnO NW were further investigated with XPS in Fig. 5. All the binding energies in the XPS analysis were corrected by referencing the C 1s line to 284.6 eV. Figure 5a exhibits the XPS full survey spectrum, from which the peaks of Zn, Sn, O, and C elements can be observed clearly. The C element might be from hydrocarbons during the synthesis process. Figure 5b,c present the high resolution spectra for Zn 2p and Sn 3d ranges, respectively. In Fig. 5b, the peaks centered at 1020.8 and 1043.9 eV are attributed to the Zn 2p_3/2_ and Zn 2p_1/2_ of Zn^2+^. The peaks appearing in Fig. 5c are located at 486.1 and 494.7 eV, which are ascribed to the Sn 3d_5/2_ and Sn 3d_3/2_ of Sn^4+^, respectively^26^,^27^. Furthermore, a peak of Sn (loss) can be seen on the higher binding energy side of Sn 3d_3/2_. Figure 5d shows a broad asymmetric curve of O 1s spectrum, which was fitted by two peaks with binding energies centered at 531.1 and 529.7 eV, indicating that two different oxygen species (O^δ−^) exist. The peak at 529.7 eV is typically ascribed to surface lattice oxygen O^2−^, and the other peak at 531.1 eV is the characteristic of surface adsorbed O^δ−^ (O_2_^−^, O^−^ etc.)^38^. It is the adsorbed O^δ−^ that react with the tested gas molecules, which improve the gas sensing performances.

**Figure 6:**
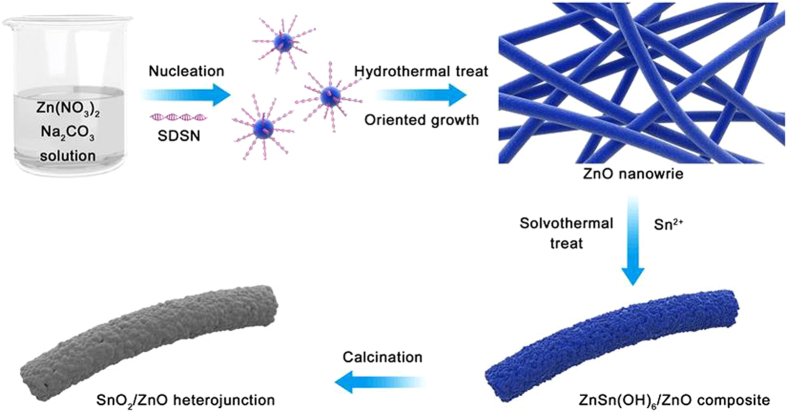
Schematic illustrations for the growth process of the as-prepared SnO_2_/ZnO NWs heterojunction.

Therefore, by combining SEM, TEM, HRTEM with EDS and XPS analysis, it can be concluded again that the fine SnO_2_ nanoparticles (*avg.* 4 nm) were successfully coated the surface of 1D ZnO NW as the heterojunctions. Based on the above results, a proposed growth process of the as-prepared SnO_2_/ZnO NW heterojunction was schematically illustrated in Fig. 6, which can be divided into three steps. Step 1 represents the formation of ZnO nanowires, and firstly excessive OH^−^ produced from the hydrolyzation of CO_3_^2−^ can react with Zn^2+^ to get Zn(OH)_2_ precipitate during aqueous solution. Under hydrothermal conditions, the formation of Zn(OH)_2_ can be accelerated in company with the dehydration into ZnO nuclei, which can grow orientedly under the assistant of structure-directing agent SDSN, and finally ZnO NWs were obtained without further treatment. In step 2, the as-prepared ZnO NW supports and Sn^2+^ were pre-dispersed in ethanol by ultrasonic treatment for 20 min to enhance their binding ability, and then a new phase of ZnSn(OH)_6_ precursor appeared after solvothermal reaction on the expense of ZnO. And in the last step 3, ZnSn(OH)_6_ precursor converted into SnO_2_ after calcination at 400 °C for 2 h, to obtain the final SnO_2_/ZnO NW product.

### Gas sensing performance

Though many SnO_2_/ZnO compositive nanomaterials have been studied for gas sensor application due to the enhanced electronic and catalytic properties^21^,^28^,^32^, there were rare reports on the n-butylamine sensing properties of 1D SnO_2_/ZnO NW. Therefore, the sensing performances of our SnO_2_/ZnO NW heterojunction have been systematically investigated, and n-butylamine was chosen as the main probe gas due to its important detection significance.

**Figure 7:**
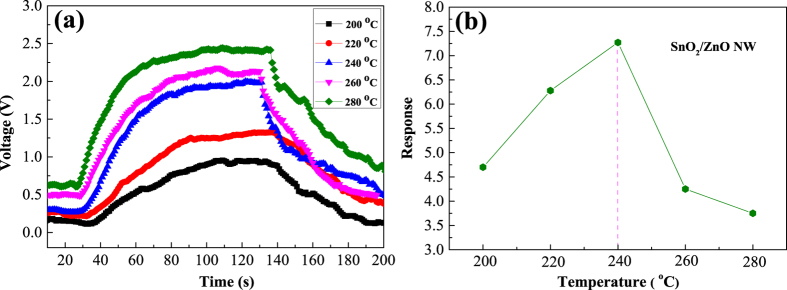
(**a**) Dynamic response–recovery curves and (**b**) the corresponding responses of the SnO_2_/ZnO NWs heterojunction-based sensor to 10 ppm n-butylamine at different OTs.

As is known, the SMO-based gas sensor is greatly influenced by the Operating Temperatures (OTs). Hence, 10 ppm n-butylamine was used as a probe gas to determine the Optimal OT. Figure 7a exhibits the dynamic response–recovery curves of the as-prepared SnO_2_/ZnO based sensor versus different OTs from 200 to 280 °C. It’s obvious that all the output voltages of the curves increase with the injection of the reductive n-butylamine vapor, while recover to the initial status after the gas is out, indicating the gas sensor is of n-type semiconductor characters. Figure 7a also clearly reveals that the gas sensor demonstrates quicker response and recovery characteristic (40 s, 80 s), and higher response amplitude at the relatively lower temperature of 240 °C. Such preferable behaviors could be attributed to the unique 1D nanowire structure, which can facilitate the mass transfer of n-butylamine molecules around the interaction region, and benefit charge carriers to traverse the barriers due to molecular recognition along the nanowires^36^.

The corresponding sensor responses at different OTs of Fig. 7a were shown in Fig. 7b, where the responses of the sensor varied with OT. When the OTs were lower than 240 °C, the response gradually rose with the increase of OT, and then reached the maximum value of 7.4 at 240 °C. However, the response decreased when increasing the OT above 240 °C. The reason may be that, the speed of gas-sensing reactions will reach balance with that of gas diffusion at a certain temperature, then the response of the gas sensor achieves the maximum^36^. Hence, 240 °C has been chosen as the Optimal OT for the SnO_2_/ZnO based sensor to carry out the following sensing tests.

**Figure 8:**
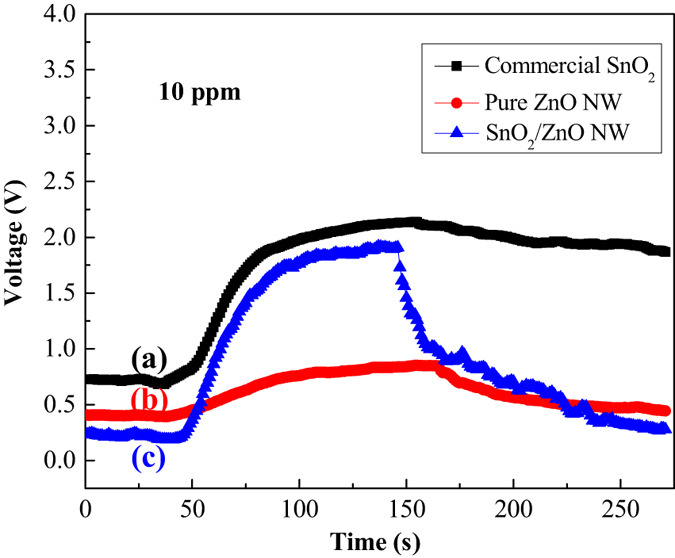
Dynamic response–recovery curves of three kinds of sensors to 10 ppm n-butylamine at 240 °C. (**a**) commercial SnO_2_, (**b**) pure ZnO NW and (**c**) SnO_2_/ZnO NW heterostructure.

Furthermore, the comparison of the response characteristics for three kinds of sensors based on pure SnO_2_, ZnO NW and SnO_2_/ZnO NW toward 10 ppm n-butylamine were carried out at 240 °C and exhibited as lines a–c in Fig. 8. It is obvious that the response amplitude of the SnO_2_/ZnO based sensor is highest than those of the pure SnO_2_ and ZnO based sensors. Besides, as shown in Fig. 8, the output voltage of line c undergoes a relatively drastic rise when n-butylamine vapour is injected in and is most rapidly restored to its base line after the gas is out, which indicates that ZnO NW based sensor possesses high response, fast response and recovery properties after the coating of SnO_2_ nanoparticles.

**Figure 9:**
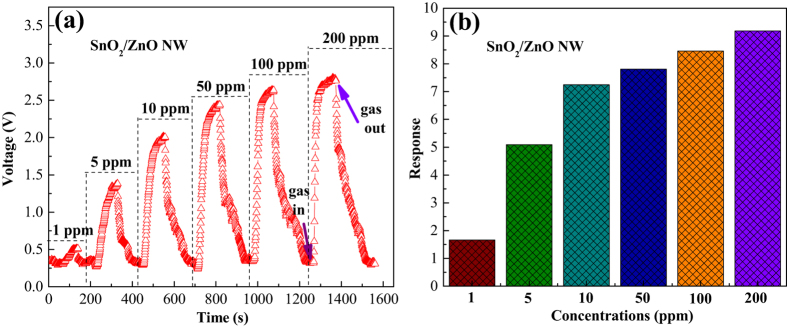
(**a**) Dynamic response–recovery curves and (**b**) the corresponding responses of the SnO_2_/ZnO heterostructure-based sensor to n-butylamine in the concentration sequence of 1, 5, 10, 50, 100, and 200 ppm at 240 °C.

To investigate the sensing ability of SnO_2_/ZnO based sensor, different concentrations of n-butylamine in the sequence of 1, 5, 10, 50, 100 and 200 ppm were tested at 240 °C, and the dynamic response-recovery curves and corresponding responses were presented in Fig. 9a,b. It is clear in Fig. 9a that the response amplitudes of SnO_2_/ZnO based sensor are significantly enhanced towards the increasing gas concentrations, meanwhile, the output voltage undergoes a drastic and then gradual upward trend when injecting the higher and higher concentrations of n-butylamine. But the speeds are more and more slowly of returning back to its initial value after the gases are out. All of the response times are within 30 s, and the relevant recovery times are no more than 55 s, indicating such SnO_2_/ZnO sensor can meet the practical demands of fast detection. Figure 9b displays the corresponding responses versus n-butylamine concentrations from Fig. 9a. It reveals clearly that the response enhances with increasing the n-butylamine vapour concentration from 1 to 200 ppm, which are 1.7, 5.1, 7.3, 7.8, 8.5 and 9.2, respectively, indicating the compositive sensor is more sensitive in lower n-butylamine concentrations.

**Figure 10:**
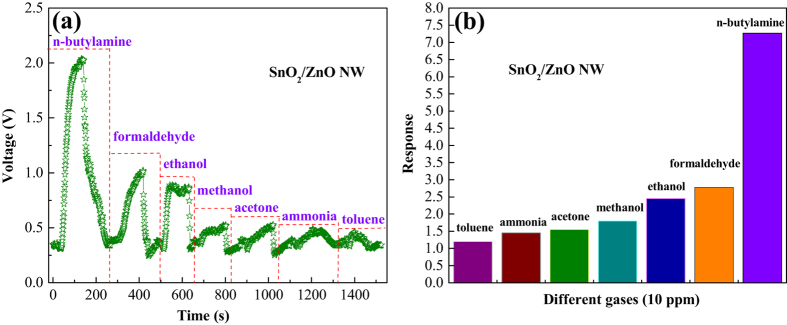
(**a**) Dynamic response–recovery curves and (**b**) the corresponding responses the SnO_2_/ZnO heterostructure-based sensor to 10 ppm different tested gases at 240 °C.

To further examine the selectivity of the SnO_2_/ZnO NW sensor, the gas sensing properties of 10 ppm other pollutant gases (toluene, ammonia, acetone, methanol, ethanol and formaldehyde) were also measured at 240 °C, which are summarized along with n-butylamine and shown in Fig. 10. The dynamic response-recovery curves of the SnO_2_/ZnO NW based sensor to different pollutant gases can be seen in Fig. 10a, and as expected, the compositive sensor exhibits obviously highest response amplitude and faster response-recovery speed to n-butylamine, then formaldehyde and ethanol are succedent. But the response trends of the gas sensor are mere and similar to the remaining gases of methanol, acetone, ammonia and toluene. The corresponding response values have been compared in Fig. 10b, which are 7.4, 2.9, 2.4, 1.8, 1.5, 1.4 and 1.2, respectively. It means that the highest response (7.4) to n-butylamine is about 2.5 times higher than that for formaldehyde (2.9), and 3.1 times higher than that for methanol (2.4), while the responses to other gases are no more than 2.

However, the reason why the compositive sensor is more sensitive to n-butylamine is still not clear. One possible reason from Kaneti *et al*.^3^ may be that, n-butylamine (C_4_H_11_N) is found to chemically adsorb on the ZnO (1 0 −1 0) surface through the formation of a bond between the N atom of C_4_H_11_N and the surface Zn atom of ZnO. Besides, the adsorption energy (E_ads_) can also indicate the possibility of adsorption, and the E_ads_ of n-butylamine (−7.30 eV) is more than those of acetone (−4.9 eV), ethanol (−7.15 eV), etc. So the response of the ZnO-based sensor toward the n-butylamine gas is higher than those to the other tested gases. Another reason could be that the different reaction activities of test gases are mainly due to their bond energies^39^, so the low C-N bond energy may accelerate reaction activity, thus can enhance the responses of gas sensors^26^,^40^. Such result indicates the potential application of our SnO_2_/ZnO NW sensor to detect n-butylamine.

**Figure 11:**
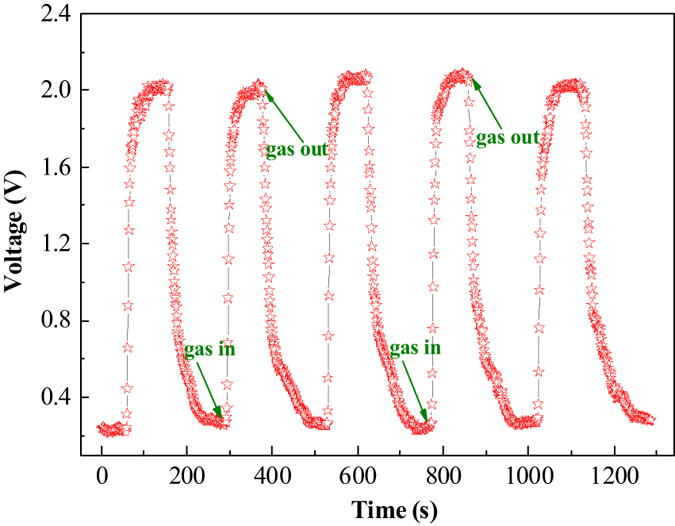
The long term stability of SnO_2_/ZnO heterostructure-based sensor to 10 ppm n-butylamine after three months at 240 °C.

In addition, Long term stability (LTS), also called reproducibility, is another important factor to evaluate the practical application of gas sensors. To investigate the LTS of SnO_2_/ZnO NW sensor, we performed five response-recovery characteristic cycles to 10 ppm n-butylamine at 240 °C after three months, as Fig. 11 has illustrated. It can be found clearly that the gas in (response) and out (recovery) curves are similar for several continuous cycles with nearly no changes in response–recovery times and response values, indicating its good reproducibility property. Moreover, it’s amazing to find that all the curves demonstrated faster response and recovery speeds (about 20 s and 40 s) than three months ago (Fig. 9) under the same test conditions, suggesting the as-prepared SnO_2_/ZnO NW heterojunction is a promising candidate for the organic amine sensing-device industries.

## Discussion

As is known, the basic sensing mechanism of N type SMOs has been interpreted by the depletion layer or space-charge model^26^,^33^,^41^. Generally speaking, the adsorption and desorption of test gas molecules on the surface of SMO-based sensing materials can lead to the reaction process of electron exchanges, which are transferred by the intermediary of surface adsorbed oxygen species O^δ−^ (O_2_^−^, O^−^ and O^2−^)^20^,^31^,^36^. Such electron transferring causes the change in the thickness of their “depleted layers” and electrical properties, thus can effectively result in the resistance/conductance changes for gas sensor devices. And the sensor sensitivity can be improved by enhancing its conductance variation.

Above is the basic working principle for pure SMO component, and similar to ZnO, the adsorption and desorption process of O^δ−^ can also occur on the surfaces of SnO_2_ nanoparticles. But the deeper working mechanism of 1D wire-like SnO_2_/ZnO NW heterostructures becomes rather complex and remains discussion. Its noticeable enhancement in the n-butylamine response may be due to the following factors. One important reason is related with the size and morphology of the sensing materials. Firstly, some early reports have proved that the sensing performances of nanoparticles are mainly decided by the relationship between particle size (D) and the Debye length (λ_D_)^42^,^43^,^44^. If the size of an SMO is close to or smaller than the Debye length, the SMO will become completely electron-depleted in air by the adsorbed O^δ−^ with high resistence. After the exposure to reducing gases, these gases react with the adsorbed O^δ−^ then the depleted electrons are released back to the SMO. Consequently, the resistance of the SMO varies substantially. Thus, a small size close to its Debye length is highly desirable to improve its sensitivity^44^. Normally, the λ_D_ of SnO_2_ is approximately 3 nm^44^,^45^, while the average D value of our outer SnO_2_ nanoparticles, the product of solvothermal reaction, is only about 4 nm, thus the “grain control effect”^42^,^43^,^44^,^45^ stands out and the small size helps greatly enhanced the sensing properties of the SnO_2_/ZnO heterostructures. Secondly, the 1D ZnO nanowire can adsorb more O_2_ molecules due to its lower tendency to agglomerate, unique electron transportation properties and larger surface-to-volume ratio than conventional nanomaterials, thus will facilitate gas diffusion through the devices and benefit the surface reactions^31^.

**Figure 12:**
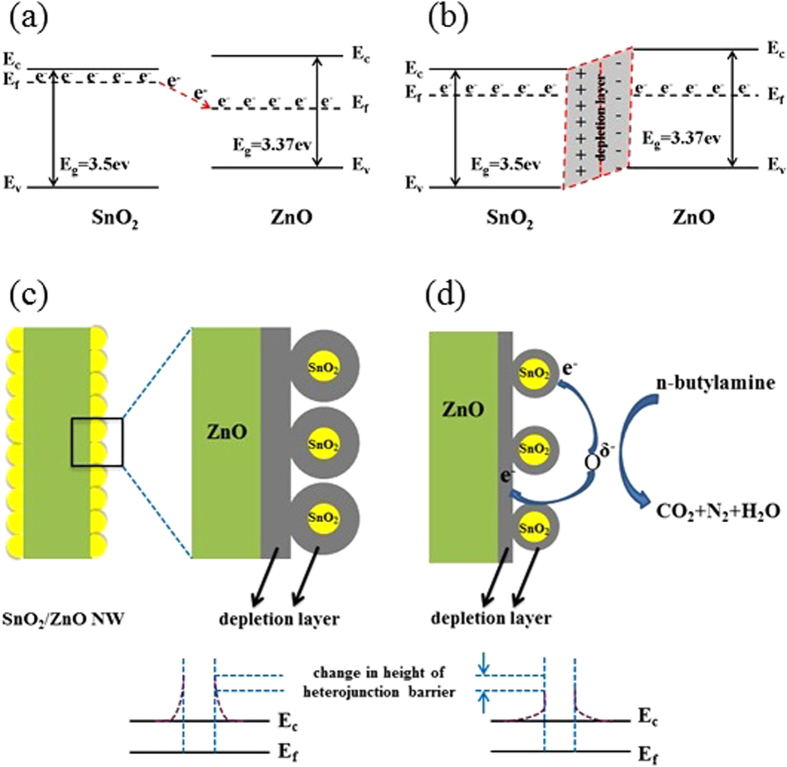
(**a,b**) The energy band structures and (**c,d**) gas sensing mechanisms of the SnO_2_/ZnO NW heterostructure sensor.

Moreover, another factor may be that, after the hydrothermal/sovolthermal treatment, a heterojunction with a potential barrier will form at the interface between the ZnO core and SnO_2_ shell, since a good interfacial contact of the two phases can be seen in Fig. 3c^26^. And the simple energy band diagrams of the heterojunction have been illustrated in Fig. 12a,b. Due to the different work functions of ZnO (5.2 eV) and SnO_2_ (4.9 eV), a unidirectional flow of electrons occurs from SnO_2_ to ZnO to equalize their Fermi levels (Fig. 12a). Then at the equilibrium state, an additional depletion layer is generated in the vicinity region of the heterojunction interfaces^36^, as illustrated in Fig. 12b, which may lead to an increase in the separation efficiency of interfacial hole-electron pairs between the two phases^46^. And then, as the surface reaction prescribes, the free electron density involved in the reactions will increase and then the dissociation of molecular O_2_ occurs easily by capturing these free electrons. Thus, a higher resistance state in air is expected in SnO_2_/ZnO heterostructures, as shown in Fig. 12c. In this case, the conductivity of the sensing material is very low, or the height of the heterojunction barriers is increased. However, just as Fig. 12d has described, when the 1D SnO_2_/ZnO heterostructure sensor is exposed to the reducing n-butylamine gas, these reducing gas moleculars can react with the adsorbed O^δ−^, and the trapped electrons will be simultaneously released back to the conduction bands of ZnO and SnO_2_, which significantly reduces the height of the potential barrier and the width of the electron depletion layer at the interfaces of the SnO_2_/ZnO NW sensor, resulting in a greatly increased conductance variation of the heterostructure. As a result, the change in the height of the heterojunction barriers in the n-butylamine atmospheres contributes to the enhanced sensing properties of the SnO_2_/ZnO NW heterojunctions^20^,^33^,^44^.

In summary, we have successfully constructed the 1D SnO_2_-coated ZnO NW heterostructures by the effective hydrothermal/solvothermal treatment followed with calcination. The obtained products were characterized by various methods to confirm that the outer layers of N-type SnO_2_ nanoparticles (*avg.* 4 nm) were uniformly distributed on the pre-synthesized n-type ZnO nanowire supports (diameter 80~100 nm, length 12~16 μm). Comparisons of the gas sensing performances among pure SnO_2_, pure ZnO NW and the as-fabricated SnO_2_/ZnO NW heterojunctions revealed that after modification, SnO_2_/ZnO NW based sensor exhibited remarkably improved response, fast response and recovery speeds, good selectivity and excellent reproducibility to n-butylamine gas, indicating it can be used as promising candidates for high-performance organic amine sensors. The enhanced gas-sensing behavior should be attributed to the small size effect of SnO_2_ nanoparticles, the unique 1D wire-like morphology of ZnO support, and the semiconductor depletion layer model as well as synergetic effect induced by the strong interfacial interaction between SnO_2_ and ZnO of the heterojunctions. The as-prepared SnO_2_/ZnO NW heterojunctions may also supply other novel applications in the fields like photocatalysis, lithium-ion batteries, waste water purification, etc.

## Materials and Methods

### Synthesis of the ZnO NWs

Reagents such as CH_3_(CH_2_)_11_SO_3_Na (SDSN), Zn(NO_3_)_2_·6H_2_O, SnCl_2_·2H_2_O, Na_2_CO_3_ and anhydrous ethanol were analytically pure and used as received without further purification. Distilled water was used throughout the experiments. The hydrothermal fabrication process of ZnO NWs was also described previously^31^,^35^ but with some changes. Typically, 1.50 g of SDSN (0.138 mol/L) and 1.52 g of Zn(NO_3_)_2_·6H_2_O (0.128 mol/L) was dissolved into 40 mL of distilled water under stirring, then 20.00 g of Na_2_CO_3_ (1.6 mol/L) was added into the above solution to form supersaturated white slurry, which was transferred to a sealed 50 mL Teflon-lined stainless autoclave and hydrothermally kept at 140 °C for 12 h. Then the white precipitation was washed with deionized water and ethanol alternately for several times, and the separated white product was dried at 60 °C for 12 h and milled to form the ZnO precursor powder.

### Synthesis of the 1D SnO_2_/ZnO NW heterojunctions

1D SnO_2_/ZnO heterojunction nanostructures were prepared via a solvothermal process. 0.042 g of the pre-synthesized ZnO NW supports were immersed into a 30 mL of ethanol solution consisting of 0.012 g of SnCl_2_·2H_2_O. Then the solution was transferred to a Teflon-lined autoclave and the solvothermal process was conducted at 120 °C for a whole day. After that, the samples were removed from the solution, washed with deionized water several times, dried at 80 °C for 20 h, and calcined at 400 °C for 2 h to gain the SnO_2_/ZnO heterojunction nanostructures.

### Characterization

The SnO_2_/ZnO samples were characterized by Powder X-ray diffraction (PXRD, Rigaku D/max-2500) in a scanning range of 10–80° (2θ), Field Emission Scanning Electron Microscope (FESEM, ZEISS EVO18), Transmission Electron Microscopy (TEM) and High Resolution TEM (HRTEM) with Energy Dispersive X-ray Spectroscopy (EDS) (Tecnai G2 F20, Holland) and X-ray Photoelectron Spectroscopy (XPS, Kratos Axis Ultra DLD spectrometer).

### Gas sensor fabrication and sensing test

Details of the gas sensor fabrication, working diagrams and test processes have also been discussed in our previous work^31^,^36^,^37^. Firstly, a proper amount of the ground sample was mixed with several drops of ethanol to form slurry, which was then coated onto the outside of the ceramic tubes with two Au electrodes and four Pt wires on each end. A Ni–Cr alloy filament was put through the ceramic tube, and used as a heater by tuning the heating voltage. Then the ceramic tube was welded onto a pedestal with six probes to obtain the final sensor unit.

The gas sensing test was carried out on a commercial WS-30A Gas-sensing Measurement System (HanWei Electronics Co., Ltd., China) with the operating temperatures (OTs) from 200 to 280 °C and the relative humidity (RH) of 40–55%. Ambient air was used as the dilution and reference gas. Target gases such as n-butylamine with calculated concentration were injected into the testing chamber by a WS-30A microsyringe. The sensor response (or sensitivity) is defined as the ratio of R_air_/R_gas_ for N type semiconductor, where Ra and Rg are the sensor resistances in air and in target gases, respectively.

## Additional Information

**How to cite this article**: Wang, L. *et al*. Construction of 1D SnO_2_-coated ZnO nanowire heterojunction for their improved n-butylamine sensing performances. *Sci. Rep.* **6**, 35079; doi: 10.1038/srep35079 (2016).
